# WAVES (Web-based tool for Analysis and Visualization of Environmental Samples)—a web application for visualization of wastewater pathogen sequencing results

**DOI:** 10.1093/bioinformatics/btad160

**Published:** 2023-04-05

**Authors:** Petr Triska, Fabian Amman, Lukas Endler, Andreas Bergthaler

**Affiliations:** Institute for Hygiene and Applied Immunology, Center for Pathophysiology, Infectiology and Immunology, Medical University of Vienna, Vienna 1090, Austria; CeMM Research Center for Molecular Medicine of the Austrian Academy of Sciences, Vienna 1090, Austria; Institute for Hygiene and Applied Immunology, Center for Pathophysiology, Infectiology and Immunology, Medical University of Vienna, Vienna 1090, Austria; CeMM Research Center for Molecular Medicine of the Austrian Academy of Sciences, Vienna 1090, Austria; Institute for Hygiene and Applied Immunology, Center for Pathophysiology, Infectiology and Immunology, Medical University of Vienna, Vienna 1090, Austria; CeMM Research Center for Molecular Medicine of the Austrian Academy of Sciences, Vienna 1090, Austria; Institute for Hygiene and Applied Immunology, Center for Pathophysiology, Infectiology and Immunology, Medical University of Vienna, Vienna 1090, Austria; CeMM Research Center for Molecular Medicine of the Austrian Academy of Sciences, Vienna 1090, Austria

## Abstract

**Motivation:**

Environmental monitoring of pathogens provides an accurate and timely source of information for public health authorities and policymakers. In the last 2 years, wastewater sequencing proved to be an effective way of detection and quantification of severe acute respiratory syndrome coronavirus 2 (SARS-CoV-2) variants circulating in population. Wastewater sequencing produces substantial amounts of geographical and genomic data. Proper visualization of spatial and temporal patterns in these data is crucial for the assessment of the epidemiological situation and forecasting. Here, we present a web-based dashboard application for the visualization and analysis of data obtained from sequencing of environmental samples. The dashboard provides multi-layered visualization of geographical and genomic data. It allows to display frequencies of detected pathogen variants as well as individual mutation frequencies. The features of WAVES (Web-based tool for Analysis and Visualization of Environmental Samples) for early tracking and detection of novel variants in the wastewater are demonstrated in an example of BA.1 variant and the signature Spike mutation S: E484A. WAVES dashboard is easily customized through the editable configuration file and can be used for different types of pathogens and environmental samples.

**Availability and implementation:**

WAVES source code is freely available at https://github.com/ptriska/WavesDash under MIT license. A demo version of this application can be accessed at: https://wavesdashboard.azurewebsites.net/.

## 1 Introduction

**Figure 1:**
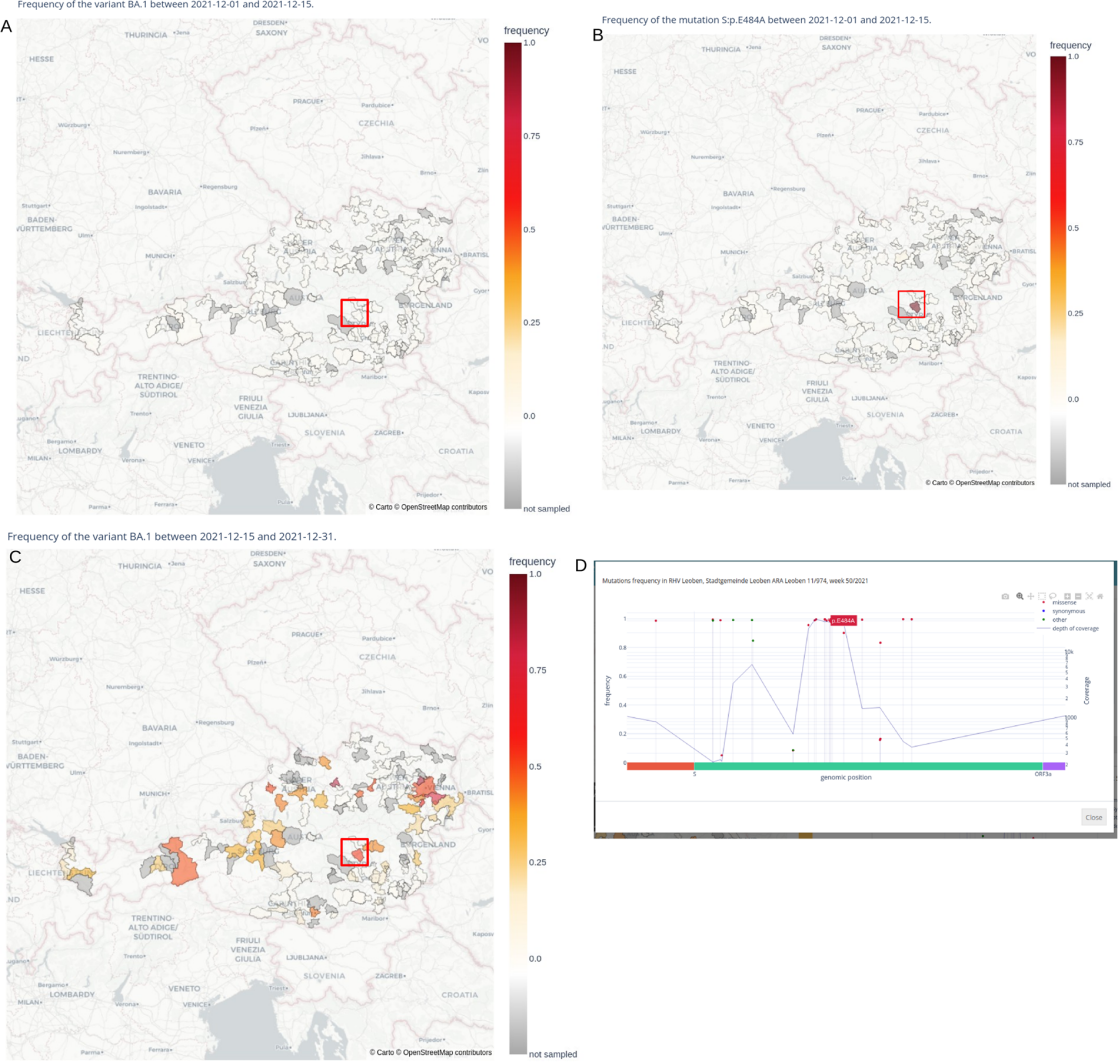
Example of use of the multi-layered data visualization with the application WAVES. (A and B) The frequency of detected BA.1 variant (A) and the BA.1-characteristic mutation S: E484A (B) in the time period from the 1st to the 15th of December 2021 in Austria was shown. While the BA.1 variant was not reliably detected in this period, the signature mutation S: E484A was already detected in the wastewater treatment plant Leoben. Two weeks later (C), the Omicron variant is detected at a high level in Leoben. The detected mutation is shown in the allele frequency plot (D)

Wastewater monitoring has been an effective method of pathogen surveillance during the SARS-CoV-2 pandemic. In countries with developed wastewater treatment infrastructure, wastewater monitoring can provide sensitive and large-scale pathogen monitoring on the population level (Amman et al. 2022). Pathogen sequencing from wastewater results in large amounts of spatial–temporal data that can be challenging to visualize and present to policymakers and the public. Here, we describe software designed for detailed visualization of the results of wastewater genome sequencing within the synoptical dashboard WAVES (Web-based tool for Analysis and Visualization of Environmental Samples). Spatial and temporal patterns in variant frequencies are particularly important for public health agencies as they help to identify introduction points of novel variants and to spot local outbreaks. Our visualization tool makes finding these patterns easier. We can illustrate this with the example of the initial spread of BA.1 variant in Austria in December 2021. While the BA.1 variant was not reliably detected (frequency > 10%) in wastewater in Austria until 19th December 2021, the characteristic Spike mutation S: E484A was detected at a high frequency more than 2 weeks earlier in sample from Leoben (Fig. 1, from raw data included in Amman et al. 2022).

## 2 Application description

### 2.1 Backend description

WAVES dashboard works as a web application. It does not require a server deployment and can be run locally on any operating system which supports Docker. The application comes with the Docker container (Merkel 2014) and can be hosted on a local machine, or it can be easily deployed in cloud services such as Azure or Amazon Web Services. The application is written in Python 3.6. (Van Rossum and Drake 2009). The backend uses the pandas (McKinney 2011) and Datatable (Stetsenko et al. 2022, retrieved from https://datatable.readthedocs.io/en/latest/index.html) packages for parsing and processing the data. The frontend is based on Plotly and Plotly Dash (Plotly Technologies Inc. 2015, retrieved from https://plot.ly.) packages for visualization of the data and for handling of user callbacks. The GitHub repository with the code can be accessed at https://github.com/ptriska/WavesDash. The application was used to produce interactive supplementary data for the recent paper by Amman et al. 2022: http://www.sarscov2-Austria.org/cemm/Austrian-sars-cov-2-ww-dashboard/.

WAVES integrates two types of data: frequencies of detected variants within individual samples, and the frequency of detected mutations within the sample. The application does not calculate either of those metrics and data must be supplied in the form of tab-separated files (tsv). Parsing of the tsv files is based on the Datatable package (Stetsenko et al. 2022). Detailed description of formatting of the input files in the readme manual located in the GitHub repository. The application parses the input files when the session is initiated. To make usage of the application smoother, the application generates precomputed data files during the first start-up. The precomputation performs a basic QC on the input files (removal of duplicates, indexing by sampling date, and sorting of the index). The update of data is done by simply removing the original data files, including the precomputed files, and replacing them with new files.

### 2.2 Frontend description

On the frontend side, WAVES consists of three vertical panels. On the left-hand side, a divide with an interactive two-layered map is located. The first layer is a CARTO background map (Carto Inc. 2022). The center point of the map and the zoom level is specified in the configuration file. The second layer consists of outlined sampling areas, which would correspond to the respective catchment areas of a given wastewater treatment plant and is defined in the user-supplied geojson file. The sampling areas are colored in choropleth style. This can be either on qualitatively (showing the most frequent variant), or quantitatively (showing the frequency of a variant or a mutation). Outlined sampling areas are interactive. When an area is selected by clicking, a plot with sequencing results for selected area will appear in the central divide. Alternatively, when a sampling area is selected from the drop-down menu in the filtering divide, the map will zoom in to show the selected area. The map also allows a time-lapse mode, in which the epidemiological situation on the map is displayed in steps corresponding to the sampling time points. The time-lapse mode requires considerable amount of memory, especially when displaying long time series. For large datasets, we recommend using geojson file with reduced resolution. Reduced geojson can be specified in the configuration file. The central divide contains two plots. The upper plot is a stacked plot showing relative frequency of each detected variant in a given sampling period. Upon selecting a sampling period by clicking at corresponding bar, a plot showing mutation frequency within a selected sample is loaded. The frequency of mutations is projected on the y-axis, while the x-axis is a genomic position of the mutation. The mutation frequency plot with the genome map can be expanded into a separate window for better readability. The right-hand panel allows for the subsetting and filtering of displayed data. Filtering is performed by selecting from drop-down menus. Filtering options contained in the drop-down forms are based on parsed data.

Detailed information on data sources, authors, and contacts can be inserted into the respective links in the main menu customizable through the configuration file. The content will be displayed in a pop-up window.

## 3 Installation

Before the application is started, the user must provide a configuration file (the template of the configuration file is present in the GitHub repository), input files in the tsv format, and a geojson file with geographic coordinates of sampling areas. The exact format of input files is described in the readme manual.

The application uses a number of python packages that need to be installed on the system prior to the start of the application. In order to simplify the installation, we provide a requrements.txt file and a prebuilt Docker image containing the whole software stack. For those who prefer to build the Docker image by themselves, we provide a Docker file located in the GitHub repository. The application starts a Flask web server which is by default accessible at localhost port 8050. The port can be changed in the configuration file.

## Data Availability

The data is available in the stated git repository.
